# Cytochrome *c* Oxidase Biogenesis and Metallochaperone Interactions: Steps in the Assembly Pathway of a Bacterial Complex

**DOI:** 10.1371/journal.pone.0170037

**Published:** 2017-01-20

**Authors:** Sonja Schimo, Ilka Wittig, Klaas M. Pos, Bernd Ludwig

**Affiliations:** 1 Institute of Biochemistry, Membrane Transport Machineries, Goethe-University, Frankfurt am Main, Germany; 2 Functional Proteomics, SFB 815 Core Unity, Faculty of Medicine, Goethe-University, Frankfurt am Main, Germany; 3 Institute of Biochemistry, Molecular Genetics, Goethe-University, Frankfurt am Main, Germany; National Research Council, ITALY

## Abstract

Biogenesis of mitochondrial cytochrome *c* oxidase (COX) is a complex process involving the coordinate expression and assembly of numerous subunits (SU) of dual genetic origin. Moreover, several auxiliary factors are required to recruit and insert the redox-active metal compounds, which in most cases are buried in their protein scaffold deep inside the membrane. Here we used a combination of gel electrophoresis and pull-down assay techniques in conjunction with immunostaining as well as complexome profiling to identify and analyze the composition of assembly intermediates in solubilized membranes of the bacterium *Paracoccus denitrificans*. Our results show that the central SUI passes through at least three intermediate complexes with distinct subunit and cofactor composition before formation of the holoenzyme and its subsequent integration into supercomplexes. We propose a model for COX biogenesis in which maturation of newly translated COX SUI is initially assisted by CtaG, a chaperone implicated in Cu_B_ site metallation, followed by the interaction with the heme chaperone Surf1c to populate the redox-active metal-heme centers in SUI. Only then the remaining smaller subunits are recruited to form the mature enzyme which ultimately associates with respiratory complexes I and III into supercomplexes.

## Introduction

The heme *aa*_3_-type cytochrome *c* oxidase (COX) is the terminal electron acceptor in the respiratory chain of mitochondria and many bacteria. It catalyzes the transfer of electrons from cytochrome *c* to molecular oxygen, a reaction that is coupled to the translocation of protons across the membrane. It thus contributes to the generation of a proton gradient, which is subsequently used to drive ATP synthesis [1, 2]. The mitochondrial enzyme consists of up to 13 different subunits (SU), with three highly conserved core SU I-III [3, 4] which are encoded by the organellar genome and represent the minimal functional entity in most bacterial oxidases as well. As the main catalytic player, COX SUI houses both the heme moieties (*a* and *a*_3_) and a copper ion (Cu_B_), which are located by one third into the depth of the membrane. SUII carries two copper ions in a mixed valence state, constituting the Cu_A_ center; it serves as the first entry point for the electron which is further shuttled to heme *a* and then to the heme *a*_3_-Cu_B_ binuclear center, where oxygen is reduced to water.

Biogenesis of this multi-subunit and multi-cofactor enzyme is a highly complex process in mitochondria, involving the coordinated expression of subunits derived from two compartmentally separated genomes, their import into the mitochondrion as well as their assembly into the holoenzyme, strictly dependent also on the synthesis, recruitment and insertion of redox-active cofactors [5–9]. Studies to understand these processes in detail and to sequentially follow those steps have suggested that COX assembly is initiated with COX SUI as a nucleation point and proceeds with the formation of distinct assembly intermediates [9, 10]. Most of these steps are assisted by assembly factors, ensuring proper progression towards a functional, correctly assembled protein complex. A majority of these chaperones has been identified by systematic analysis of COX biogenesis defects in *Saccharomyces cerevisiae*, revealing more than 30 such auxiliary proteins [8, 11]. However, many of these are implicated in transcriptional and translational regulation or involved in early assembly steps, often poorly conserved between higher eukaryotes [12, 13]. Among the assembly factors with the highest degree of sequence conservation are those involved in the synthesis or incorporation of cofactors, some of them are even part of the terminal oxidase operon in bacteria [14, 15], emphasizing their importance and suggesting that the core assembly processes share still today, from bacteria to eukaryotes, similar mechanistic features.

Focussing on the metallation of COX SUI, it is worth mentioning that heme *a* is an exclusive cofactor of terminal oxidases. This cofactor is synthesized in two steps from heme *b* in the mitochondrion, involving two membrane-bound enzymes. First, heme *o* synthase (Cox10 in eukaryotes, CtaB in prokaryotes) transfers a farnesyl diphosphate to heme *b* [16], which is subsequently oxidized by heme *a* synthase (Cox15 in eukaryotes, CtaA in prokaryotes) to form heme *a* [17]. Whilst heme *a* biosynthesis is essential for COX assembly [18, 19], the exact mechanism of cofactor insertion and players involved remains unresolved. A recent study in mitochondria suggests that oligomerization of Cox15 may be important for both heme *a* biosynthesis and subsequent delivery to maturing COX [20], as Cox15 has also been found to be associated with Cox SUI containing assembly intermediates and with the assembly factor Shy1p (Surf1c in prokaryotes; [21]). The latter is a well-known COX biogenesis factor, whose functional loss is connected to Leigh syndrome in humans, a severe neurodegenerative disorder [22–24]. Even though the yeast homologue Shy1p is one of the best studied Surf1 homologues and found to interact with COX assembly intermediates [21, 25–27], studies in bacteria established its role as a heme *a* binding protein and its involvement in the hemylation of COX SUI [14, 28, 29].

The Cu_B_ site formation in COX SUI is dependent on the assembly factor Cox11 (termed CtaG in prokaryotes), substantially supported by studies in *Rhodobacter sphaeroide*s and *S*. *cerevisiae* [30–32]. Recently, a physical association of CtaG and COX SUI was confirmed in *Paracoccus denitrificans* [33].

The Sco proteins Sco1 and Sco2 are implicated in the metallation of the Cu_A_ center in COX SUII. Whilst Sco1 is generally accepted to play an active role in this process since a direct interaction with SUII was confirmed in both yeast and mammalian cells [34–38], the ultimate function of Sco2 is still under debate. In humans Sco2 may act as a thiol-disulfide oxidoreductase for Sco1 and thus is indirectly involved in the maturation of SUII [39, 40].

Bacterial oxidases are far simpler in structure compared to their mitochondrial counterparts, comprising only a basic set of subunits whose expression and assembly is dependent on a single genetic system and a limited number of key biogenesis factors. Therefore, *P*. *denitrificans*, one of the closest evolutionary relatives to present-day mitochondria [41], is a well-suited model to study fundamental processes of COX biogenesis.

Here we present additional insight into the assembly pathway of maturing bacterial cytochrome *c* oxidase and its interplay with COX specific metallochaperones. Blue native electrophoresis in combination with either Western blot analysis or complexome profiling and pull-down assays allow us to identify and characterize the composition of four distinct COX SUI assembly intermediates. Our data suggest an early association of this subunit with CtaG, followed by the interaction with Surf1c, the subsequent association with SUII, III and IV, and the final insertion of the holoenzyme into respiratory supercomplexes.

## Materials and Methods

### Chemicals

*n*-dodecyl-β-D-maltoside (DDM; >99.5%) was purchased from GLYCON Biochemicals (Luckenwalde, Germany) and digitonin (99.9%) from Roth (Karlsruhe, Germany). Protein A/G-coated magnetic beads, the PageRuler prestained protein ladder #26616 and the amine-reactive crosslinker dithiobis(succinimidyl) propionate (DSP) were purchased from Thermo Scientific (Waltham, MA, USA).

### *Paracoccus denitrificans* strains

The parental strain used in this study was Pd1222 (DSM413 derivative, Rif^r^, Sp^r^, enhanced conjugation frequencies, m^-^; [42]). In strain FA2 (Pd1222, Δ*surf*1c::Kan^r^, Rif^r^; [15]) the *surf*1c gene was replaced by a kanamycin resistance cassette introduced by homologous recombination. Both alleles of cytochrome *c* oxidase subunit I are replaced in strain MR31 (Pd1222, Δ*cta*DI::Km^r^, Δ*cta*DII:Tc^r^; [43]) by kanamycin and tetracycline resistance genes. In strain ST4 (Δ*cta*::*neo*) most of the *cta* operon from *cta*C to *cta*E was replaced by a kanamycin resistance gene [44], see also Table 1.

**Table 1:** Genotype and sources of *P*. *denitrificans* strains.

Strain	Genotype	Strain description	source
Pd1222	DSM413 derivative, Rif^r^, Sp^r^, enhanced conjugation frequencies, m^-^	WT	[42]
FA2	Pd1222, Δ*surf*1c::Kan^r^, Rif^r^	deletion of Surf1c gene	[15]
MR31	Pd1222, Δ*cta*DI::Km^r^, Δ*ctaD*II:Tc^r^	deletions of both COX SUI genes	[43]
ST4	Δ*cta*CBGE::Km^r^	large deletion affecting COX SUII, SUIII, CtaB and CtaG	[44]

### *In vivo* crosslinking of intact cells, and membrane preparation

*P*. *denitrificans* cultures were grown to exponential phase and washed cells were crosslinked essentially as in [33] with DSP at a final concentration of 1 mM. Cells were disrupted using a Constant System cell disrupter (Constant Systems Ltd) at 1.85 kbar, followed by membrane preparation as described previously [28]. The protein concentration was determined using a modified Lowry protocol [45, 46].

### Blue native gel electrophoresis

For blue native polyacrylamide gel electrophoresis (BN-PAGE) untreated membranes were solubilized with digitonin, mixed with appropriate loading buffer and applied to a 3.5–18% gradient BN gel (140 μg protein per lane) with dimensions of 0.07 x 8.3 x 7.3 cm, essentially as described in [47]. For second-dimension SDS-PAGE, lanes were cut out, incubated in 1% SDS for 10 min at room temperature and run on a 10% SDS Tricine gel [48]. The separated complexes were either analyzed by silver-staining or by immunoblotting [28].

For complexome profiling, BN-PAGE was performed alike, employing a 3–18% BN gradient gel [47] with dimensions of 0.16 x 14 x 14 cm. As native protein standard, digitonin-solubilized bovine heart mitochondria were used [49].

### Complexome profiling and data analysis

Complexome profiling (quantitative mass spectrometry) was performed essentially as described [50, 51] with the following modifications: peptides were loaded on a C18 reversed-phase precolumn (Zorbax 300SB-C18, Agilent Technologies) followed by separation on in-house packed 2.4 μm Reprosil C18 resin (Dr. Maisch GmbH) picotip emitter tip (diameter 100 μm, 15 cm long, New Objectives) using a gradient from mobile phase A (4% acetonitrile, 0.1% formic acid) to 30% mobile phase B (80% acetonitrile, 0.1% formic acid) for 20 min followed by a second gradient to 70% mobile phase B with a flow rate of 300 nl/min. Reference proteome set of *P*. *denitrificans* Uniprot (Download November 2015, 5019 entries) was used to identify peptides and proteins with a false discovery rate (FDR) of 1%.

Abundance profiles were generated by NOVA software [52] using intensity-based absolute quantification (IBAQ) values from MaxQuant. IBAQ values of proteins from gel lane fractions were normalized to maximum appearance.

Bovine heart mitochondria were used as mass ladder [53]. The corresponding slice number of the maximum appearance of mitochondrial complex IV (210 kDa), complex III dimer (480 kDa), complex V (620 kDa), complex I (1000 kDa) and respiratory supercomplex containing complex I, III dimer and one copy of complex IV (1650 kDa) was used for mass calibration. The equation [f(x) = 18228*e^(0.1076x), R^2^ = 0.9848] obtained by exponential regression was used to calculate the native masses of each slice.

### Immunoprecipitation using magnetic beads

In case of untreated membranes (non-crosslinked), digitonin was used at a detergent/protein ratio of 8 g/g to solubilize 5 mg of membranes in 0.5 ml of 50 mM Tris/HCl (pH 7.2) for 1 h at 4°C. Crosslinked membranes were solubilized with DDM (detergent to protein ratio of 2 g/g) and cleared by ultracentrifugation at 100 000 x g for 1h and 4°C. A suspension of protein A/G magnetic beads (25 μl) was washed with buffer (20 mM Tris/HCl pH 7.2, 0.08% digitonin for untreated membranes; 20 mM Tris/HCl pH 7.2, 0.02% DDM for crosslinked membranes), incubated for 1 h at room temperature with antiserum for immunoprecipitation and subsequently incubated with the clarified solubilisate (1 h, room temperature). Unbound material was separated, using a magnetic stand, and beads were washed twice with 0.5 ml IP buffer (20 mM Tris/HCl pH 7.2, 50 mM NaCl, 0.08% digitonin for untreated membranes; 20 mM Tris/HCl pH 7.2, 150 mM NaCl, 0.02% DDM in case of crosslinked membranes), followed by the elution of bound proteins in 100 μl 0.1 M glycine (pH 2) and immediate neutralization with 50 mM Tris/HCl (pH 7.5). As control unrelated antiserum was used. The eluted fractions were separated by 12% SDS PAGE and subsequently analyzed by immunoblotting.

### Western blot analysis

For immunostaining, proteins were transferred to nitrocellulose membrane and treated with rabbit anti-COX (mainly recognizing COX SUI and II;[33]), anti-CtaG [33], anti-Surf1c or anti-CtaA antisera according to standard procedures; for detection, protein A alkaline phosphatase (Merck, Darmstadt, Germany) was used. The specificity of the employed antisera is documented in the S2 Fig.

## Results

Biogenesis of cytochrome *c* oxidase is a highly complex process, involving both the recruitment of subunits as well as the incorporation of redox cofactors delivered by specific chaperones. We used two independent detection methods, immunostaining and mass spectrometry (MS), to identify intermediary assembly complexes during biogenesis of the heme *aa*_3_-type terminal oxidase in *P*. *denitrificans*. First protein interactions were probed by two-dimensional gel electrophoresis and immunoprecipitation and further analyzed by complexome profiling, providing insight on additional COX biogenesis factors and their roles in the assembly process.

### COX assembly intermediates and interacting metallochaperones detected by immunostaining

Membranes from *P*. *denitrificans*, prepared from wildtype (WT) and deletion strain cells, were solubilized with the mild detergent digitonin and separated in the first dimension by BN-PAGE, ensuring the preservation of weak protein-protein-interactions. A subsequent denaturing SDS-PAGE run in the second dimension was followed by Western blotting and used for the analysis of interacting components from the first dimension gel. Western blots were probed with antibodies directed against COX (recognizing mainly SUI and SUII), CtaG, Surf1c and CtaA.

**Fig 1:**
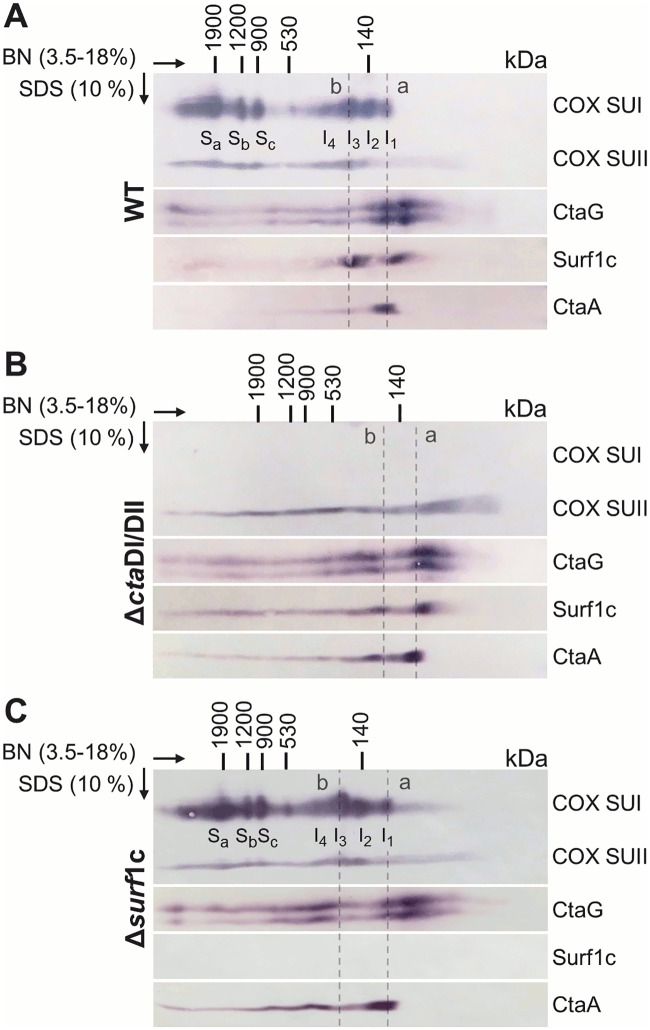
Two-dimensional BN/SDS-PAGE and Western blot analysis of COX assembly complexes. 140 μg membranes prepared from *P*. *denitrificans* cells from strains (A) WT, (B) MR31 (Δ*cta*DI/DII), (C) FA2 (Δ*surf*1c) were solubilized with digitonin at a detergent/protein ratio of 4 g/g and subjected to a first dimension 3.5–18% gradient BN-PAGE. A gel strip was excised, soaked in 1% SDS and placed on top of a 10% SDS Tricine gel for separation in the second dimension. Proteins were transferred to a nitrocellulose membrane and analyzed by Western blotting with polyclonal antibodies directed against COX (recognizing SUI and SUII), CtaG, Surf1c or CtaA. To fully ascertain the positional alignment between different Western blots, critical blots were either re-stained with a second antibody of interest, or initially developed with a mixture of different antibodies (not shown). The approximate molecular masses have been assigned based on a molecular weight marker control and a previous mass estimation for molecular complexes in *P*. *denitrificans* [49]. The positions of the COX SUI subassemblies I_1-4_ as well as the three supercomplexes S_a-c_ (nomenclature as in [49]) are indicated. In (A) the dotted line a shows COX SUI (62.4 kDa) and CtaG (21.4 kDa; known to migrate as double band on SDS PAGE [33]) on a vertical, whereas line b marks co-migration of COX SUI, SUII (28 kDa), CtaG, Surf1c (24.6 kDa) and less prominently of CtaA (41.8 kDa); in panel B these assembly complexes are not observed, however the position of lines a and b is maintained as in (A) and (C) for comparison purposes.

Staining of COX SUI (Fig 1A) shows four distinct spots with approximate sizes of 100 (I_1_), 140 (I_2_), 200 (I_3_) and 300 (I_4_) kDa, revealing well-defined COX subassemblies, see below. Co-migration of COX SUII is observed first on the level of subassemblies I_2_ and more dominant on I_3_, suggesting a late entry point into the assembly line. Both COX SUI and SUII are also found in the three distinct supercomplexes at 900 (S_c_), 1200 (S_b_) and 1900 (S_a_) kDa as previously reported [49]. These supercomplexes are associations of oxidase with either respiratory complex III and cytochrome *c*_552_ (S_c_: III_4_IV_2_, S_b_: III_4_IV_4_) or of complex III, complex I and cyt *c*_552_ (S_a_: I_1_III_4_IV_4_; S1, S3 and S4 Figs). Upon deletion of both COX SUI gene loci (MR31, deletions in *cta*DI/DII; see Table 1) supercomplex formation is severely impaired and SUII is found spread from the low to the high molecular weight region (Fig 1B and S1, S3 and S4 Figs).

The copper chaperone CtaG is generally believed to mediate copper transfer into COX SUI [7]. Our data show two well-defined spots in the first dimension between around 60 and 120 kDa for CtaG (Fig 1A). We assume that the first spot (60–80 kDa) represents a higher homo-oligomeric state of CtaG (monomer 21.4 kDa; [12, 54]), since under normal growth conditions, only a minority of this chaperone should be actively engaged in interactions with newly synthesized COX subunits. Thus, this low molecular band may correspond to “idling” CtaG chaperone copies, whereas the second signal at higher molecular weight reveals a co-migration of the CtaG with the 62.4 kDa SUI (COX subcomplex I_1_, dotted line a) and may hint at a genuine interaction between the chaperone and this oxidase subunit. To further support this observation, we analyzed the CtaG migration behaviour in an oxidase SUI deletion strain, demonstrating a clear shift of relative staining intensity towards the low molecular weight signal (Fig 1B). Additionally, staining of CtaG is also observed at higher molecular weight between 200 and 500 kDa (see Fig 1A) and even more pronounced in either mutant background (Fig 1B and 1C), hinting at associations of CtaG in complexes, even then the target subunit is missing.

The chaperone Surf1c is implicated in the insertion of heme into COX SUI [29]. Fig 1A shows two distinct spots at approximately 70 kDa and with higher staining intensity between 150 and 200 kDa. Similarly as for CtaG, we interpret the lowest molecular weight signal as the “idling” portion of Surf1c which is not actively involved in oxidase biogenesis interactions. The higher molecular weight spot coincides with COX-subcomplex I_3_ and is significantly diminished in the oxidase SUI deletion strain (Fig 1B), hinting at an association between COX SUI and Surf1c (monomer 24.6 kDa); at the same time, this observation would also suggest that a COX chaperone biogenesis complex may exist to some extent (see Discussion) even when the target subunit is missing. Further, this same subassembly I_3_, running in the first dimension at approximately 200 kDa, comprises distinct signals for COX SUII (28 kDa) and CtaG (21.4 kDa) as indicated by the dashed line b. Upon deletion of Surf1c (Fig 1C), we find that COX SUI, SUII and CtaG distribute very similarly in the first dimension gel, including the supercomplexes S_a-c_.

The membrane-bound enzyme CtaA catalyzes the final step in the formation of heme *a*, a redox-active cofactor found exclusively in terminal oxidases [55]. We therefore asked whether CtaA may play a pivotal role in oxidase assembly as well. For CtaA in a WT background we observe one dominant band migrating in the first dimension at approximately 90–120 kDa and most probably representing a higher oligomeric state of CtaA (monomer 41.8 kDa; [20]). A second, weaker signal is detected at higher molecular weight at approximately 200 kDa, which becomes more dominant in both the Surf1c and oxidase SUI deletion strains and may hint at associations with oxidase subunits or other COX assembly factors.

Taken together, we present a likely sequence of events for the maturation of SUI, as represented by subcomplexes I_1_ to I_4_, showing both the interactions with metallochaperones and the entry of further COX subunits.

### Direct interaction between oxidase and assembly factors Surf1c and CtaG

To further support the association of Surf1c, CtaG and CtaA with oxidase assembly intermediates, we performed a series of co-immunoprecipitations (co-IP), employing anti-COX, anti-CtaG, anti-Surf1c and anti-CtaA as bait antibodies and unrelated serum as control. Membranes were either prepared from exponentially growing untreated (non-crosslinked) cells or cells that were additionally crosslinked with DSP *in vivo* to covalently stabilize transient protein interactions. For each set of membranes, prepared from WT or deletion strains, pull-downs were conducted in parallel, incubating solubilized membranes with antibody-coated protein A/G magnetic beads. Beads were washed carefully to remove unspecific associations, specifically bound proteins were eluted with glycine (pH 2), immediately neutralized, and analyzed by Western blotting.

**Fig 2:**
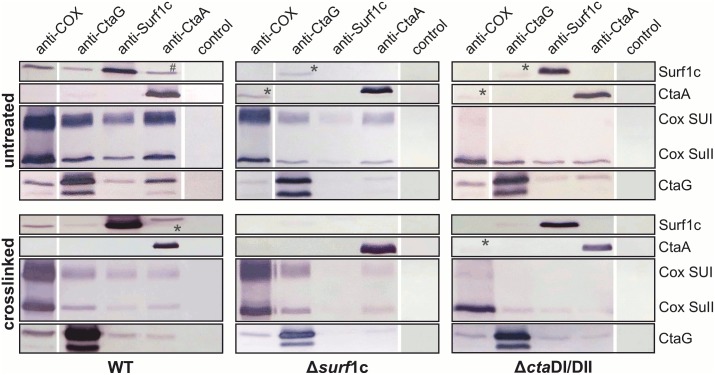
Pull-down assays indicating interactions between COX, Surf1c and CtaG. Interactions between COX, Surf1c, CtaG and CtaA were assayed by immunoprecipitation and subsequent Western blot analysis. Solubilized membranes from untreated (top) or crosslinked cells (bottom), including WT and deletion strains FA2 (Δ*surf*1c) and MR31 (Δ*cta*DI/DII), were used for Co-IP experiments with protein A/G magnetic beads, as described in the experimental procedures. Eluates were separated by 12% SDS PAGE, blotted and probed against Surf1c, CtaA, COX SUI, COX SUII and CtaG. As bait antibody anti-COX (recognizing SUI and II), anti-CtaG, anti-Surf1c, anti-CtaA or a control serum was employed. The hashtag indicates a non-reproducible interaction and the asterisk designates unspecific staining (different molecular weight bands compared to control).

Fig 2 shows that all bait antibodies efficiently precipitate their respective target protein as expected. Using anti-COX as bait antibody (Fig 2, WT), not only COX subunits are pulled out, but also CtaG (as previously shown [33]) and, moreover, Surf1c. The reciprocal IP confirms these results, demonstrating for the first time a direct interaction between Surf1c and oxidase in the bacterial model system. Interestingly, the interaction between oxidase and the COX SUI specific Cu_B_ metallochaperone CtaG, is not exclusively mediated by COX SUI, but to some extent also by COX SUII, as documented in the oxidase SUI deletion strain. Furthermore, the association between CtaG and oxidase is independent of the heme chaperone Surf1c (Fig 2, FA2), however immunoprecipitation of Surf1c led to the co-IP of limited quantities of CtaG, and *vice versa*, indicating that these two proteins do interact with each other (Fig 2, WT).

The reciprocal interactions observed between oxidase and its assembly chaperones were further confirmed by *in vivo* crosslinking (Fig 2, crosslinked). This approach allows to covalently stabilize genuine protein interactions and to challenge isolated membranes in a much more stringent way during solubilization of membranes and in all subsequent washing steps, employing the more efficient detergent DDM instead of the rather mild detergent digitonin. Signals for the crosslinked samples are generally weaker, since chemical crosslinking as such and in particular in intact cells is not very efficient. Hence, under these harsher experimental conditions, yields for interacting complexes are lower.

For heme *a* synthase CtaA, there is no clear indication for either an association with oxidase or with the COX assembly factors CtaG and Surf1c. Even though Co-IP of COX or CtaG with CtaA is shown, the reciprocal experiment does not confirm this interaction.

### Extending our view using a combined BN-PAGE-MS approach

**Fig 3:**
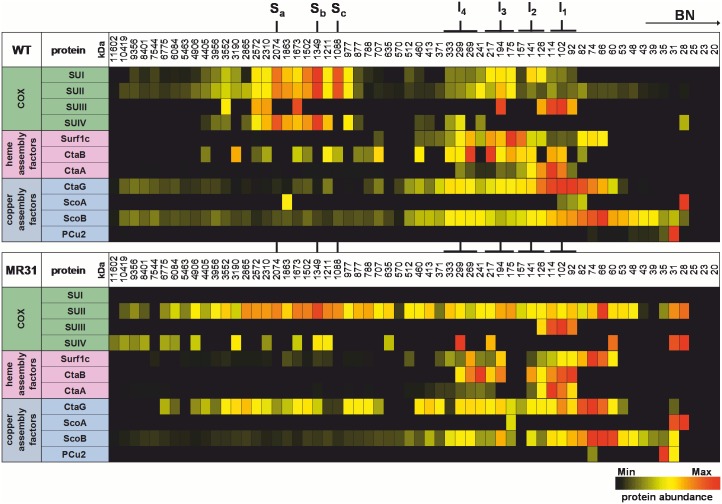
Heat map illustration of COX subunits and biogenesis factors identified by complexome profiling. 140 μg of membranes prepared from WT (top panel) or oxidase SUI double deletion strain MR31 (Δ*cta*DI/DII; lower panel) were solubilized with detergent (digitonin:protein 2:1), separated by BN-PAGE and subsequently analyzed by quantitative MS. The position of supercomplexes (S_a-c_) and COX SUI assembly intermediates (I_1-4_) is indicated. For each identified protein, the data are normalized to maximum appearance over all 60 gel slices in each sample.

**Fig 4:**
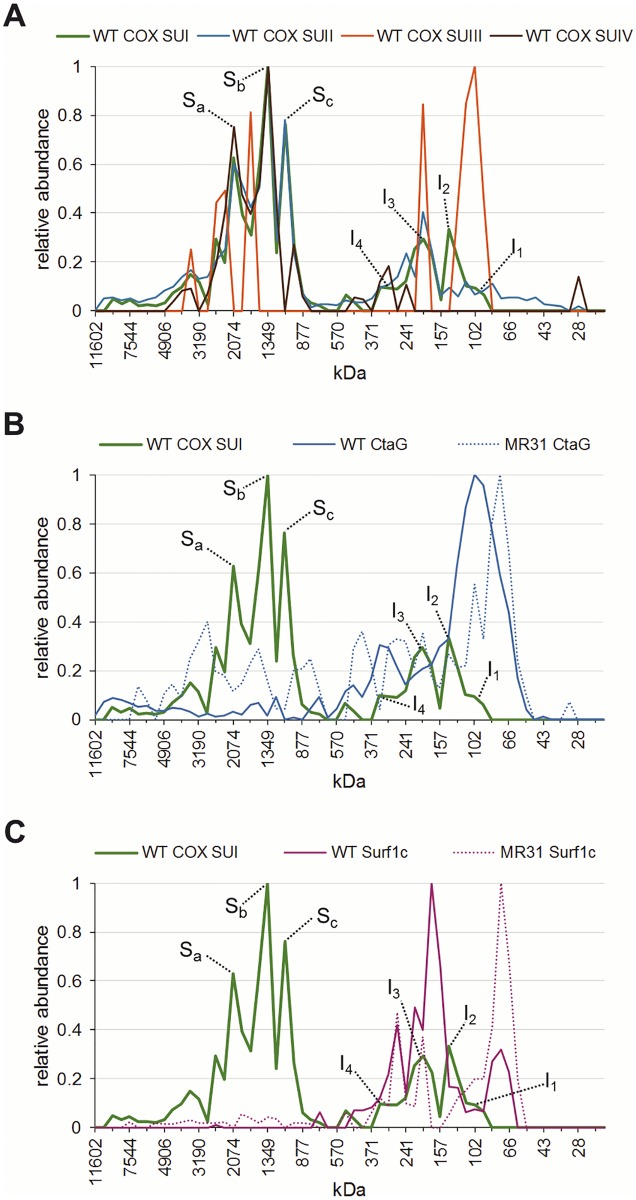
Migration profiles of COX and metallochaperones CtaG and Surf1c obtained by complexome profiling. One-dimensional BN-PAGE of digitonin-solubilized membranes from *P*. *denitrificans* was analyzed by MS-based complexome profiling. Each panel shows the relative abundance of identified proteins plotted against the apparent molecular mass after BN-PAGE separation. Corresponding heat maps are shown in Fig 3. All profiles are presented as indicated in the legend. (A) Migration profiles of all four COX subunits in a WT background are shown and the positions of subcomplexes I_1-4_ and supercomplexes S_a-c_ are indicated. (B) Comparison of CtaG migration pattern in membranes prepared from WT or strain MR31, deleted in both COX SUI gene loci, together with the COX SUI WT migration profile. (C) The migration profile of Surf1c is shown for WT and oxidase SUI deletion strain MR31 together with the migration pattern of WT COX SUI.

Complexome profiling combines the advantages of BN-PAGE analysis, separating native protein complexes at high resolution, with MS for the identification of the underlying protein complexes and subunits [50]. In this study we used this method as a complementary approach to map COX assembly intermediates. After separation of digitonin-solubilized membranes by BN-PAGE, the gel was cut into 60 slices, subjected to in-gel trypsin digestion, followed by nano liquid chromatography-MS. A heat map illustration of selected proteins and corresponding migration profiles confirmed our previous finding that COX SUI is found in four distinct assembly intermediates (I_1_-I_4_; Figs 3 and 4A). Additionally, we find that both SUII (28 kDa) and SUIII (30.8 kDa) enter the assembly line only at the stage of subcomplex I_3_, and assume this latter SU to also populate I_4_ as well. Due to its high hydrophobicity often leading to recovery problems [51] on the one hand, and the low overall protein amounts found for I_4_, we may fall below the detection limit here (see Figs 1A and 3A). SUIII is also found between 90 and 120 kDa in WT and in the oxidase SUI deletion strain; however, this position may represent only a storage pool of newly synthesized oligomeric SUIII.

Co-migration of SUIV with the COX core complex is observed shortly after the formation of subassembly I_3_ at approximately 240 kDa and more dominantly at 300 kDa, corresponding to subcomplex I_4_. Interestingly, the largest proportion of COX subunits is found in the supercomplexes S_c_, S_b_ and S_a_. Consistent with our previous results supercomplex formation is lost altogether in the MR31 strain [49] but also in strain ST4 (Δ*cta*CBGE::Km^r^; [44]), which carries, amongst others, deletions in the structural genes for SUII and SUIII, but was previously characterized to lack the SUI protein as well (Fig 3 and S1, S3 and S4 Figs).

Concerning the COX specific copper assembly factors, the complexome analysis shows co-migration of CtaG with COX SUI subcomplex I_1_ and to a minor extent with the subassemblies I_2_-I_4_. In the absence of COX SUI a clear shift of CtaG migration behaviour is observed from approximately 100 kDa (I_1_) to 75 kDa (Figs 3 and 4B), supporting our previous finding of an early association of CtaG with COX SUI. The *P*. *denitrificans* copper chaperone ScoB is the main player in the metallation of the Cu_A_ site of COX SUII [56]. ScoB (monomer 22.6 kDa) is widely distributed, with the highest proportion migrating at 66 kDa, which could be a complex of dimeric ScoB and COX SUII (28 kDa). This notion is supported by the dominant migration behaviour at around 66 kDa of COX SUII in the oxidase SUI deletion strain (Fig 3) and may hint to COX SUII being metallated prior to association with COX SUI.

For the proteins involved in heme *a* synthesis and hemylation of COX SUI our complexome data show migration of heme *a* synthase CtaA between 90 and 140 kDa. A similar distribution of CtaA is found in the MR31 and ST4 strains (Fig 3 and S3 Fig), suggesting that CtaA may be present in a higher homo-oligomeric state (see [20]) or at least not distinctly associated with COX SUI (Fig 3). In contrast, for Surf1c a clear association with subcomplex I_2_ and I_3_ is observed, where the strongest signal is visible at around 165 kDa, a position where COX SUII and SUIII do not yet interact with SUI. These Surf1c signals are lost in the absence of COX SUI (strain MR31, Δ*cta*DI/DII), and Surf1c dominantly migrates at lower molecular weight between 60 and 80 kDa (Figs 3 and 4C).

## Discussion

Defects in the biogenesis pathway of mitochondrial COX are associated with severe respiratory deficiencies. Distinct stages are passed during assembly of this multisubunit enzyme, involving both the timely association of subunits as well as the integration of their crucial redox-active metal centers. In eukaryotes more than 30 auxiliary factors have been identified that assist the expression and assembly process, of which only a limited set is conserved in bacteria such as *P*. *denitrificans*, where they are mainly implicated in either cofactor synthesis or delivery [7, 12, 13, 57]. So far, however, a comprehensive understanding of the sequential assembly steps and of cofactor incorporation is still lacking. In this study we focus on the identification of intermediary COX assembly states towards a functional holoenzyme using the bacterium *P*. *denitrificans* as a model system. Based on our data, we suggest a scenario for a sequential assembly of oxidase subunits together with their accompanying metallochaperone interactions.

**Fig 5:**
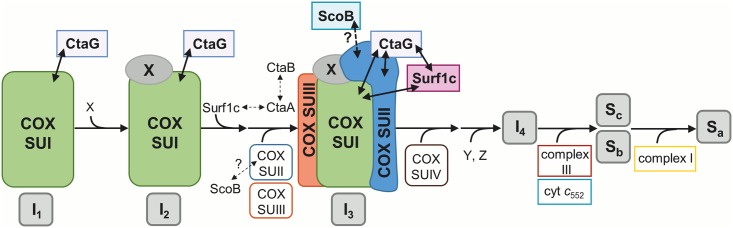
Proposed model of the sequential COX assembly pathway in *P*. *denitrificans*. COX subunits, respiratory chain complexes and assembly factors are depicted schematically. Direct interactions confirmed by our data are indicated by solid arrows, and presumed interactions by dashed arrows, respectively. Newly translated COX SUI interacts with copper chaperone CtaG to form subcomplex I_1_. This complex evolves into subcomplex I_2_, characterized by an extra mass X of approximately 40 kDa. Formation of subcomplex I_2_ is followed by the interaction with Surf1c. Direct interactions between Surf1c and CtaA as well as between CtaB and CtaA are indicated by dashed arrows. After association of Surf1c, COX SUII and SUIII are recruited, leading to formation of subcomplex I_3_. Addition of SUIV leads to formation of the holoenzyme complex, which subsequently associates with subunits of respiratory chain complexes III and I, passing through the supercomplex assembly intermediate I_4_ and ultimately ends up in supercomplexes S_c_, S_b_ and S_a_.

Three main experimental approaches were taken to gain insight into the COX assembly pathway, heavily relying on the use of mild, non-destructive techniques to preserve weak protein interactions. Initially, intermediary complexes and associations were identified using gel electrophoresis in combination with Western blot analysis. In a second step, we selectively co-immunoprecipitated and enriched these complexes, to confirm specific interactions, and finally we conducted a complexome analysis to extend our view towards all proteins likely involved in oxidase biogenesis. Our data now allow us to follow the assembly/metallation processes for COX, starting from newly synthesized SUI all the way to the final insertion of the holoenzyme into supercomplexes (Fig 5).

We find that COX SUI progresses through at least four intermediate states (I_1_-I_4_), which are discernible by immunostaining after 2D BN/SDS PAGE and are further confirmed by complexome profiling. From co-migration/association behaviour of COX SUII and SUIII, these two subunits apparently enter the stage only at subcomplex I_3_, followed by the addition of SUIV (I_3_-I_4_), and the subsequent association with respiratory chain complexes I and III into supercomplexes (S_a-c_; Fig 4). Additionally, we can define subcomplex-specific associations with the *Paracoccus* metallochaperones CtaG and Surf1c (Figs 1 and 4).

CtaG is generally believed to be involved in the insertion of a copper ion into the Cu_B_ site of COX SUI [25, 30], and a direct interaction between these two players was suggested earlier [33]. In addition, we now provide strong evidence for an early association of dimeric CtaG (monomer 21.4 kDa; [54, 58, 59]) with newly synthesized COX SUI (62.4 kDa) to form subcomplex I_1_ (≈100 kDa; Figs 1 and 4). Based on this finding it is tempting to speculate that metallation of the Cu_B_ site is the initial step in the assembly line, likely mediated in a co-translational manner [33]. As CtaG remains associated, even though to a minor extent, with later COX assembly intermediates (see Figs 1A and 3A), an additional role as a docking or recruitment factor is conceivable. If the assembly progress is distorted in either mutant background, we speculate that a subfraction of each chaperone (such as in the case of CtaG) does remain attached, for an extended time window, to these subcomplexes. As we also observe an interaction of CtaG with SUII in the pulldown experiments (see Fig 2, Δ*cta*DI/DII), we may even ascribe this chaperone the role of a basic scaffold for a COX biogenesis complex, attracting other required chaperones as well. Prior to association of the heme chaperone Surf1c, subcomplex I_1_ evolves into subcomplex I_2_, characterized by an extra mass of approximately 40 kDa (in total 140 kDa). This suggests that a so far unknown factor(s) participates in the complex formation.

Surf1c is predominantly found in a larger COX SUI complex, ranging between 160 and 180 kDa which also overlaps with subcomplex I_3_ (200 kDa), as confirmed by a shift of migration behaviour in the oxidase SUI deletion strain (Figs 1 and 4). Recruitment of Surf1c may be mediated by both COX SUI and/or CtaG as an interaction between all three proteins was unequivocally confirmed in the WT strain by Co-IP experiments (Fig 2). In prokaryotes, Surf1c has been strongly linked to the insertion of both heme cofactors into COX SUI, where a significant loss of the heme moiety was observed upon Surf1 deletion [14, 15]: we previously suggested a functional role of Surf1c as both a recruitment factor and as a filter, not only retrieving the heme *a* molecule from its site of synthesis, CtaA, but also discriminating this cofactor over heme o before its insertion into SUI [29]. In the COX holoenzyme, the hydrophobic farnesyl side chain of the heme *a*_3_ entity is embedded between SUI and SUII [60], suggesting that heme *a* insertion in SUI likely occurs before association with SUII. Consistent with our present data, we conclude that heme insertion takes place in a Surf1c-assisted manner, just prior to formation of subcomplex I_3_ and the concomitant association of COX SUII and III. Upon deletion of Surf1c, we observe no significant accumulation of COX SUI assembly intermediates as judged from quantifying relative spot distributions by densitometry (not shown): missing hemylation of SUI seems to go largely unnoticed during further COX biosynthesis steps. As the enzyme activity of isolated oxidase was reported to be highly diminished when purified from a Surf1c deletion mutant, an assembled four-subunit complex obviously does form *in vivo* which, however, lacks activity for the fact that the heme binding sites in SUI remain unpopulated [15, 61]. Yet, an alternative but less efficient heme insertion pathway independent of Surf1c, must be operative, since a fraction of fully cofactor-assembled and therefore active oxidase was reported for Surf1 deletions in all organisms studied so far [15, 23, 24]. In this context heme *a* synthase CtaA may be discussed as immediate alternative donor in the hemylation of COX SUI. Previous studies in *S*. *cerevisiae* demonstrated a direct interaction between Cox15 (yeast homologue of CtaA) and Shy1p (homologue of Surf1c) in a biogenesis complex and proposed a more direct role of Cox15 as heme *a* insertase [21]. Based on our results, we cannot confirm an association of CtaA with either COX assembly intermediates or Surf1c, however from *in vitro* experiments there is no doubt that Surf1c and CtaA do interact [61]. We therefore conclude that this interaction must be highly transient and evades our present approach. However, based on our complexome profiling (data not shown), it is worth mentioning that Surf1q [15], involved in the delivery of heme *a* to the *ba*_3_ quinol oxidase, is not associated with COX assembly intermediates, again emphasizing the specificity of the interaction between the *aa*_3_-type terminal oxidase and its corresponding heme chaperone, see [15, 33].

The assembly of COX SUII (28 kDa) and SUIII (30.8 kDa) is observed on the level of subcomplex I_3_ (200 kDa). In addition to the COX subunits (121 kDa) this subassembly comprises both the metallochaperones Surf1c (24.6 kDa) and CtaG (21.4 kDa). In this context, CtaG may act as physical mediator for the recruitment of COX SUII, as we observe a direct interaction between CtaG and SUII in our pull-down experiments. Studies with human Sco1 and Sco2 deficient cell lines proposed that Cu_A_ center formation is required prior to association of SUII with SUI [62]. Our complexome data (see Fig 3) show that ScoB (22.6 kDa) co-migrates with all subcomplexes I_1_-I_4_, however is mainly found between 60 and 80 kDa, where it is also co-localized with COX SUII when assembly is impaired due to the absence of SUI. This finding may be discussed in support of the above mentioned hypothesis, however metallation of SUII after assembly with SUI cannot be completely ruled out. Our MS data are fully consistent with the earlier observation that neither the second Sco homologue ScoA nor the copper chaperone PCu are involved in this COX biogenesis step [56, 63], clearly confirming that ScoB is the main player in *P*. *denitrificans*.

Interestingly, we found that prior to association of SUIII this subunit is also present in another complex between 90 and 120 kDa, which accumulates when the main assembly partner COX SUI is missing. We therefore assume that *de novo* translated SUIII is kept in an assembly-competent state, readily available for the assembly process. Co-migration of SUIV is first observed shortly after formation of subcomplex I_3_, suggesting that this subunit is added as the final component to complete formation of the holoenzyme. Importantly COX biogenesis does not stop at this stage, as COX subunits are predominantly found in supercomplexes together with respiratory chain complexes I and III. Therefore, based on complexome profiling, we assume that subcomplex I_4_ may represent an early association of functional oxidase with subunit components of complex I.

On the basis of our observations, we suggest a sequential biogenesis model (see Fig 5) for this bacterial COX:

1. COX assembly initiates with SUI, progressing through at least 3 intermediates (I_1_-I_3_) to complete formation of the holoenzyme.
2. Together with COX SUI, CtaG forms the first subcomplex I_1_, suggesting that copper insertion is an early event during oxidase biogenesis possibly independent of heme cofactors.
3. Surf1c enters the assembly line prior to formation of subcomplex I_3_ and we hypothesize that Surf1c-mediated heme insertion precedes the addition of SUII and SUIII.
4. Recruitment of SUII may be mediated by assembly factor CtaG, as a direct interaction between SUII and CtaG was observed. Both SUII and SUIII may enter the assembly process in parallel, leading to formation of subcomplex I_3_.
5. Assembly proceeds with the addition of SUIV, completing formation of an active four-subunit enzyme.
6. Without significant accumulation in membranes, biogenesis of the holo-form progresses further, leading to the formation of distinct supercomplexes additionally comprising respiratory chain complexes I and III as well as cyt *c*_552_.

## Supporting Information

S1 Fig Separation of solubilized membranes by 2D BN/SDS-PAGE and subsequent silver staining. 140 μg membranes prepared from WT, strain FA2 (Δ*surf*1c), strain MR31 (Δ*cta*DI/DII) or strain ST4 (Δ*cta*CBGE) were solubilized with digitonin (digitonin:protein 4:1) and subjected to a first dimension 3.5–18% gradient BN-PAGE. A gel strip was excised and layered on top of a 10% SDS Tricine gel for separation in the second dimension, followed by silver staining. Masses in the first dimension gel were assigned according to (Stroh A, Anderka O, Pfeiffer K, Yagi T, Finel M, Ludwig B, et al. Assembly of respiratory complexes I, III, and IV into NADH oxidase supercomplex stabilizes complex I in *Paracoccus denitrificans*. The Journal of Biological Chemistry. 2004;279(6):5000–7.). Supercomplexes S_a_, S_b_ and S_c_ run at apparent molecular masses of 1900, 1200 and 900 kDa in the first dimension gel and are separated into their subunit components in the denaturing second dimension SDS gel. Whilst these complexes are present in membranes obtained from WT and strain FA2 (indicated by black bars), they are missing in membranes prepared from strain MR31 and ST4 (expected molecular weight positions of hypothetical supercomplexes S_a-c_ are indicated by grey bars).
(PDF)

S2 Fig Specificity of antisera used in pull-down experiments. In order to check antibody specificity, the following samples were loaded onto a 12% SDS-PAGE and blotted onto nitrocellulose membrane for subsequent Western blot analysis. Blots were probed with the indicated polyclonal rabbit antisera (anti-COX, anti-CtaG (see also Gurumoorthy P, Ludwig B. Deciphering protein-protein interactions during the biogenesis of cytochrome *c* oxidase from *Paracoccus denitrificans*. The FEBS journal. 2015;282(3):537–49), anti-Surf1c and anti-CtaA (custom immunization, Cambridge Research Biochemicals)) and developed by incubation with protein A-alkaline phosphatase as described in Materials and Methods: M, prestained protein ladder (Thermo Fisher Scientific, 26616); 1, 100 μg *P*. *denitrificans* (Pd1222) wildtype membranes; 2, 0.1 μg purified cytochrome *c* oxidase from *P*. *denitrificans*; 3, 0.1 μg purified CtaA from *P*. *denitrificans* (His_6_-tagged construct); 4, 0.1 μg purified Surf1c from *P*. *denitrificans* (His_10_-tagged construct); 5, 0.1 μg purified CtaG from *P*. *denitrificans* (His_6_-tagged construct). Protein bands at appropriate sizes are indicated by arrows (panel anti-CtaA, lane 3: lower molecular weight bands may represent proteolytic digestion fragments).
(PDF)

S3 Fig Complexome profiles obtained from *P*. *denitrificans* WT and deletion strains. Membranes (140 μg) obtained from *P*. *denitrificans* WT (A) and deletion strains MR31 (B) and ST4 (C) were solublized with digitionin with a detergent/protein ratio of 2 g/g and and separated by BN-PAGE. The gel lanes were cut into 60 identical slices, trypsin digested and analyzed by liquid chromatography tandem-mass spectrometry. The complexome profiles of respiratory chain complexes and oxidase assembly factors are shown as heat map illustration. For each identified protein, intensity-based absolute quantification (IBAQ) values were normalized to maximum appearance over all 60 slices. Oxidative phosphorylation complexes from bovine heart mitochondria were used for mass calibration. The position of supercomplexes S_a-c_ and assembly intermediates I_1-4_, as found in the WT strain, is indicated (black bars in WT, grey bars in strain MR31 and ST4).
(PDF)

S4 Fig Migration profiles of respiratory chain complexes. Digitonin-solubilized membranes (140 μg) from *P*. *denitrificans* were separated by BN-PAGE and subsequently analyzed by complexome profiling. The average relative abundance value of each complex (taking all its subunits into account) is plotted against the apparent molecular mass in the BN gel. All profiles are coloured as shown in the legend. (A) The migration pattern of respiratory chain complexes I, III and IV, as well as of cyt *c*_552_ is shown in a WT background. Co-migration in the supercomplexes S_a-c_ is indicated, where complex I is only present in supercomplex S_a_. Membranes from strain MR31 (B) or strain ST4 (C) do not exhibit supercomplexes.
(PDF)

## Abbreviations

BN: blue native
Co-IP: co-immunoprecipitation
complex I: NADH:ubiquinone oxidoreductase
complex III: ubiquinol:cytochrome *c* oxidoreductase
complex V: ATP synthase
COX: cytochrome *c* oxidase or complex IV
cyt *c*_552_: cytochrome *c*_552_
DDM: *n*-dodecyl-β-D-maltoside
DSP: dithiobis(succinimidyl)propionate
IBAQ: intensity-based absolute quantification
MS: mass spectrometry
PAGE: polyacrylamide gel electrophoresis
SU: subunit
WT: wildtype
