# Supplementary material for: Cytochrome c Oxidase Biogenesis and Metallochaperone Interactions: Steps in the Assembly Pathway of a Bacterial Complex
Source: PLoS One. 2017 Jan 20;12(1):e0170037. doi: 10.1371/journal.pone.0170037 (PMC5249081; doi:10.1371/journal.pone.0170037)
Supplement: S1 Fig — 140 μg membranes prepared from WT, strain FA2 (Δsurf1c), strain MR31 (ΔctaDI/DII) or strain ST4 (ΔctaCBGE) were solubilized with digitonin (digitonin:protein 4:1) and subjected to a first dimension 3.5–18% gradient BN-PAGE. A gel strip was excised and layered on top of a 10% SDS Tricine gel for separation in the second dimension, followed by silver staining. Masses in the first dimension gel were assigned according to (Stroh A, Anderka O, Pfeiffer K, Yagi T, Finel M, Ludwig B, et al. Assembly of respiratory complexes I, III, and IV into NADH oxidase supercomplex stabilizes complex I in Paracoccus denitrificans. The Journal of Biological Chemistry. 2004;279(6):5000–7.). Supercomplexes Sa, Sb and Sc run at apparent molecular masses of 1900, 1200 and 900 kDa in the first dimension gel and are separated into their subunit components in the denaturing second dimension SDS gel. Whilst these complexes are present in membranes obtained from WT and strain FA2 (indicated by black bars), they are missing in membranes prepared from strain MR31 and ST4 (expected molecular weight positions of hypothetical supercomplexes Sa-c are indicated by grey bars). (PDF) [file pone.0170037.s001.pdf]

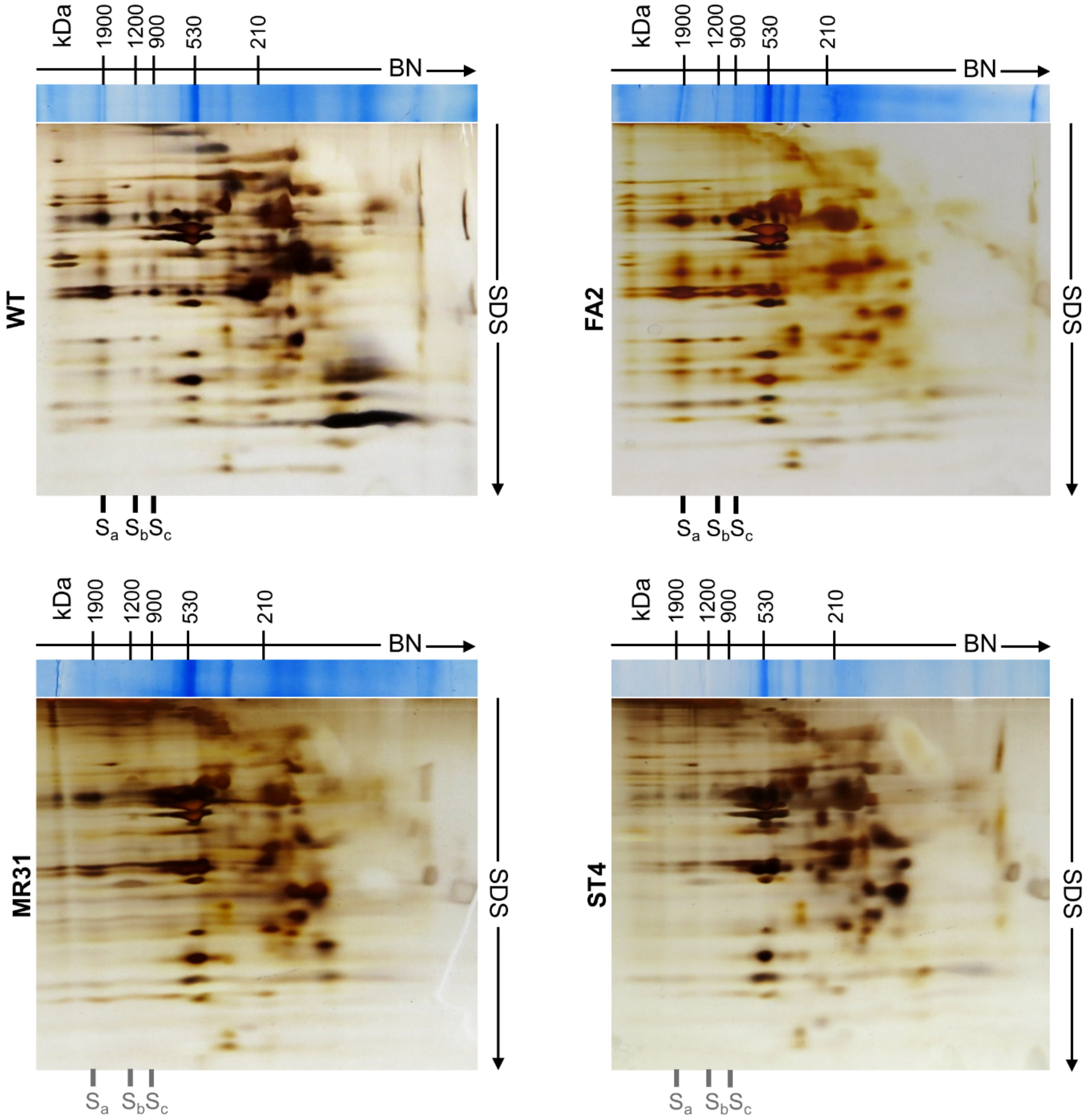

S1 Fig. Separation of solubilized membranes by 2D BN/SDS-PAGE and subsequent silver staining. 140 µg membranes prepared from WT, strain FA2 (Δ*surf*1c), strain MR31 (Δ*cta*DI/DII) or strain ST4 (Δ*cta*CBGE) were solubilized with digitonin (digitonin:protein 4:1) and subjected to a first dimension 3.5-18 % gradient BN-PAGE. A gel strip was excised and layered on top of a 10 % SDS Tricine gel for separation in the second dimension, followed by silver staining. Masses in the first dimension gel were assigned according to (Stroh A, Anderka O, Pfeiffer K, Yagi T, Finel M, Ludwig B, et al. Assembly of respiratory complexes I, III, and IV into NADH oxidase supercomplex stabilizes complex I in *Paracoccus denitrificans*. The Journal of Biological Chemistry. 2004;279(6):5000-7.). Supercomplexes S_a, S_b and S_c run at apparent molecular masses of 1900, 1200 and 900 kDa in the first dimension gel and are separated into their subunit components in the denaturing second dimension SDS gel. Whilst these complexes are present in membranes obtained from WT and strain FA2 (indicated by black bars), they are missing in membranes prepared from strain MR31 and ST4 (expected molecular weight positions of hypothetical supercomplexes $S_{a\text{-}c}$ are indicated by grey bars).
