# Supplementary material for: Cytochrome c Oxidase Biogenesis and Metallochaperone Interactions: Steps in the Assembly Pathway of a Bacterial Complex
Source: PLoS One. 2017 Jan 20;12(1):e0170037. doi: 10.1371/journal.pone.0170037 (PMC5249081; doi:10.1371/journal.pone.0170037)
Supplement: S2 Fig — In order to check antibody specificity, the following samples were loaded onto a 12% SDS-PAGE and blotted onto nitrocellulose membrane for subsequent Western blot analysis. Blots were probed with the indicated polyclonal rabbit antisera (anti-COX, anti-CtaG (see also Gurumoorthy P, Ludwig B. Deciphering protein-protein interactions during the biogenesis of cytochrome c oxidase from Paracoccus denitrificans. The FEBS journal. 2015;282(3):537–49), anti-Surf1c and anti-CtaA (custom immunization, Cambridge Research Biochemicals)) and developed by incubation with protein A-alkaline phosphatase as described in Materials and Methods: M, prestained protein ladder (Thermo Fisher Scientific, 26616); 1, 100 μg P. denitrificans (Pd1222) wildtype membranes; 2, 0.1 μg purified cytochrome c oxidase from P. denitrificans; 3, 0.1 μg purified CtaA from P. denitrificans (His6-tagged construct); 4, 0.1 μg purified Surf1c from P. denitrificans (His10-tagged construct); 5, 0.1 μg purified CtaG from P. denitrificans (His6-tagged construct). Protein bands at appropriate sizes are indicated by arrows (panel anti-CtaA, lane 3: lower molecular weight bands may represent proteolytic digestion fragments). (PDF) [file pone.0170037.s002.pdf]

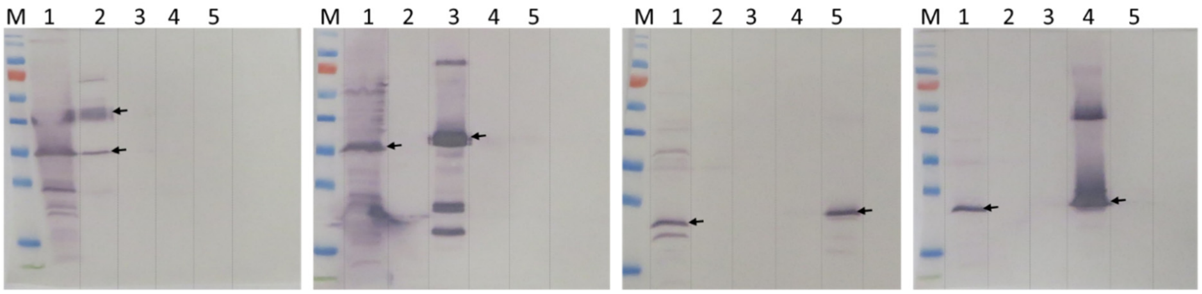

S2 Fig. Specificity of antisera used in pull-down experiments. In order to check antibody specificity, the following samples were loaded onto a 12 % SDS-PAGE and blotted onto nitrocellulose membrane for subsequent Western blot analysis. Blots were probed with the indicated polyclonal rabbit antisera (anti-COX, anti-CtaG (see also Gurumoorthy P, Ludwig B. Deciphering protein-protein interactions during the biogenesis of cytochrome *c* oxidase from *Paracoccus denitrificans*. The FEBS journal. 2015;282(3):537-49), anti-Surf1c and anti-CtaA (custom immunization, Cambridge Research Biochemicals)) and developed by incubation with protein A-alkaline phosphatase as described in Materials and Methods: M, prestained protein ladder (Thermo Fisher Scientific, 26616); 1, 100 µg *P. denitrificans* (Pd1222) wildtype membranes; 2, 0.1 µg purified cytochrome *c* oxidase from *P. denitrificans*; 3, 0.1 µg purified CtaA from *P. denitrificans* (His_6-tagged construct); 4, 0.1 µg purified Surf1c from *P. denitrificans* (His_{10}-tagged construct); 5, 0.1 µg purified CtaG from *P. denitrificans* (His_6-tagged construct). Protein bands at appropriate sizes are indicated by arrows (panel anti-CtaA, lane 3: lower molecular weight bands may represent proteolytic digestion fragments).
