# Supplementary material for: Cytochrome c Oxidase Biogenesis and Metallochaperone Interactions: Steps in the Assembly Pathway of a Bacterial Complex
Source: PLoS One. 2017 Jan 20;12(1):e0170037. doi: 10.1371/journal.pone.0170037 (PMC5249081; doi:10.1371/journal.pone.0170037)
Supplement: S3 Fig — Membranes (140 μg) obtained from P. denitrificans WT (A) and deletion strains MR31 (B) and ST4 (C) were solublized with digitionin with a detergent/protein ratio of 2 g/g and and separated by BN-PAGE. The gel lanes were cut into 60 identical slices, trypsin digested and analyzed by liquid chromatography tandem-mass spectrometry. The complexome profiles of respiratory chain complexes and oxidase assembly factors are shown as heat map illustration. For each identified protein, intensity-based absolute quantification (IBAQ) values were normalized to maximum appearance over all 60 slices. Oxidative phosphorylation complexes from bovine heart mitochondria were used for mass calibration. The position of supercomplexes Sa-c and assembly intermediates I1-4, as found in the WT strain, is indicated (black bars in WT, grey bars in strain MR31 and ST4). (PDF) [file pone.0170037.s003.pdf]

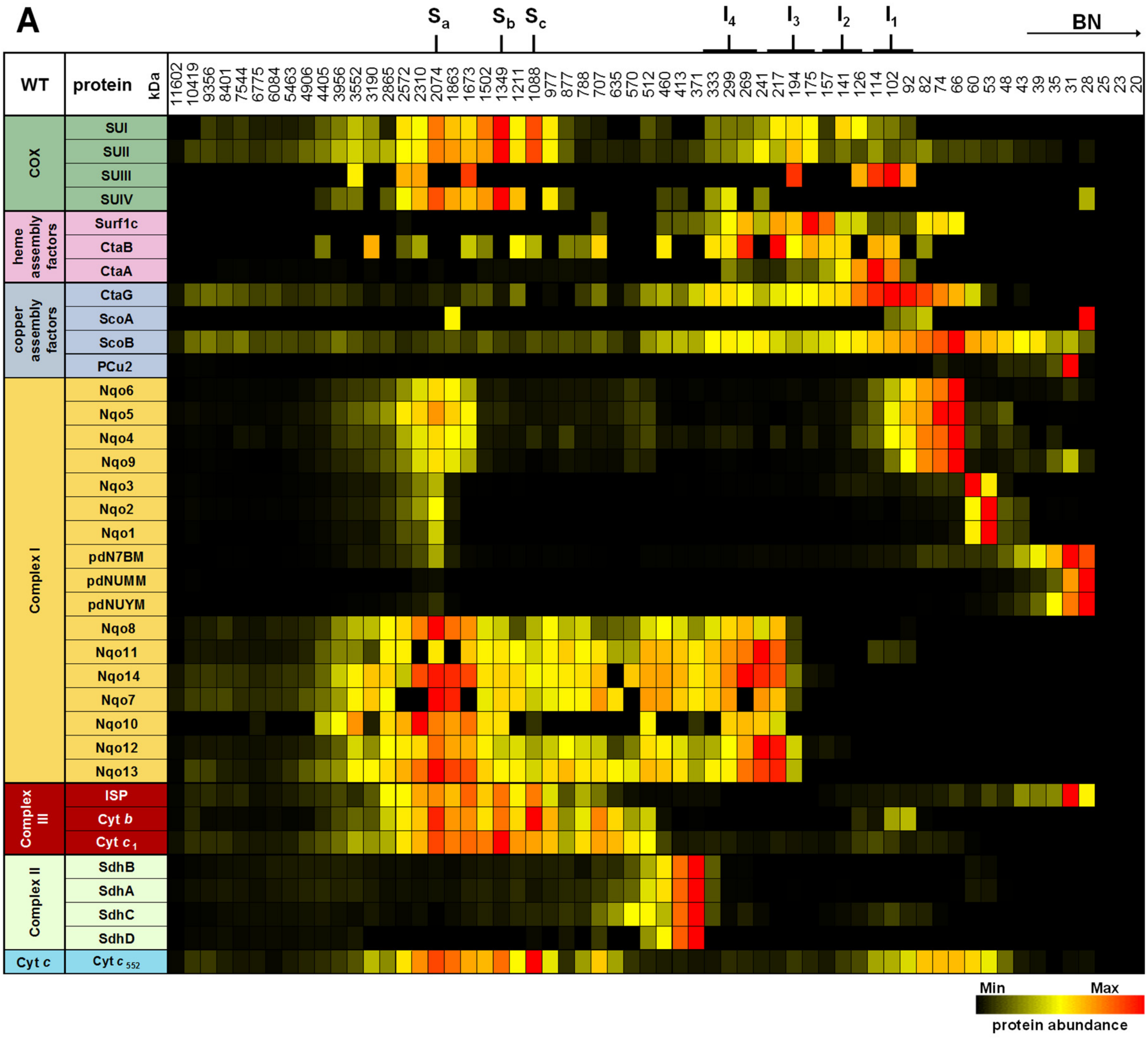
A
Sa
Sb
Sc
I4
I3
I2
I1
BN
WT
protein
kDa
11602 10419 9356 8401 7544 6775 6084 5463 4906 4405 3956 3552 3190 2865 2572 2310 2074 1863 1673 1502 1349 1211 1088 977 877 788 707 635 570 512 460 413 371 333 299 269 241 217 194 175 157 141 126 114 102 92 82 74 66 60 53 48 43 39 35 31 28 25 23 20
COX
SUI
SUII
SUIII
SUIV
heme assembly factors
Surf1c
CtaB
CtaA
copper assembly factors
CtaG
ScoA
ScoB
PCu2
Complex I
Nqo6
Nqo5
Nqo4
Nqo9
Nqo3
Nqo2
Nqo1
pdN7BM
pdNUMM
pdNUYM
Nqo8
Nqo11
Nqo14
Nqo7
Nqo10
Nqo12
Nqo13
Complex III
ISP
Cyt b
Cyt c1
Complex II
SdhB
SdhA
SdhC
SdhD
Cyt c
Cyt c552
Min
Max
protein abundance

B

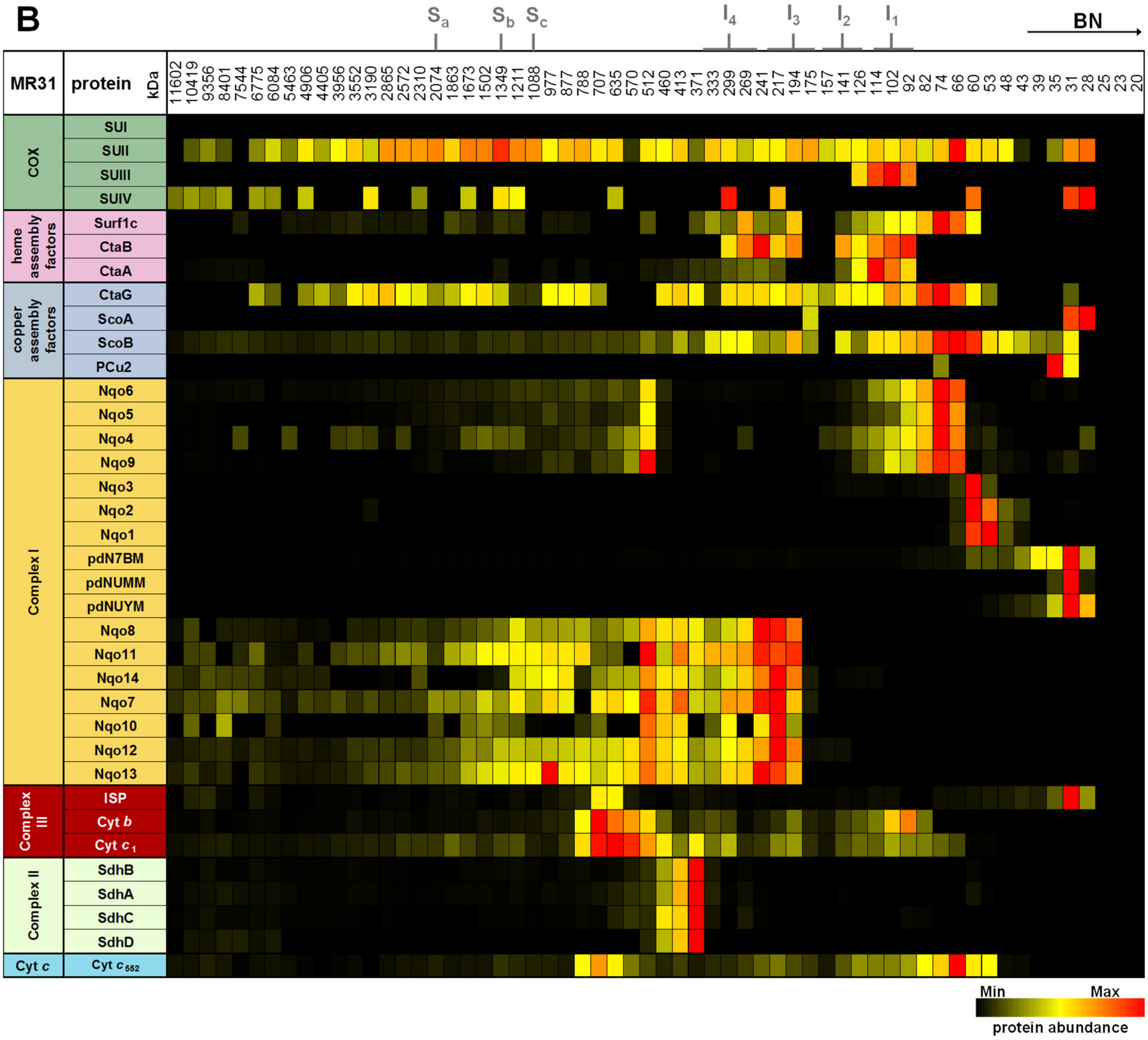

MR31
protein
kDa
BN
Sa Sb Sc
I4 I3 I2 I1
11602 10419 9356 8401 7544 6775 6084 5463 4906 4405 3956 3552 3190 2865 2572 2310 2074 1863 1673 1502 1349 1211 1088 977 877 788 707 635 570 512 460 413 371 333 299 269 241 217 194 175 157 141 126 114 102 92 82 74 66 60 53 48 43 39 35 31 28 25 23 20
COX
SUI
SUII
SUIII
SUIV
heme assembly factors
Surf1c
CtaB
CtaA
copper assembly factors
CtaG
ScoA
ScoB
PCu2
Complex I
Nqo6
Nqo5
Nqo4
Nqo9
Nqo3
Nqo2
Nqo1
pdN7BM
pdNUMM
pdNUYM
Nqo8
Nqo11
Nqo14
Nqo7
Nqo10
Nqo12
Nqo13
Complex III
ISP
Cyt b
Cyt c1
Complex II
SdhB
SdhA
SdhC
SdhD
Cyt c
Cyt c552
Min
Max
protein abundance

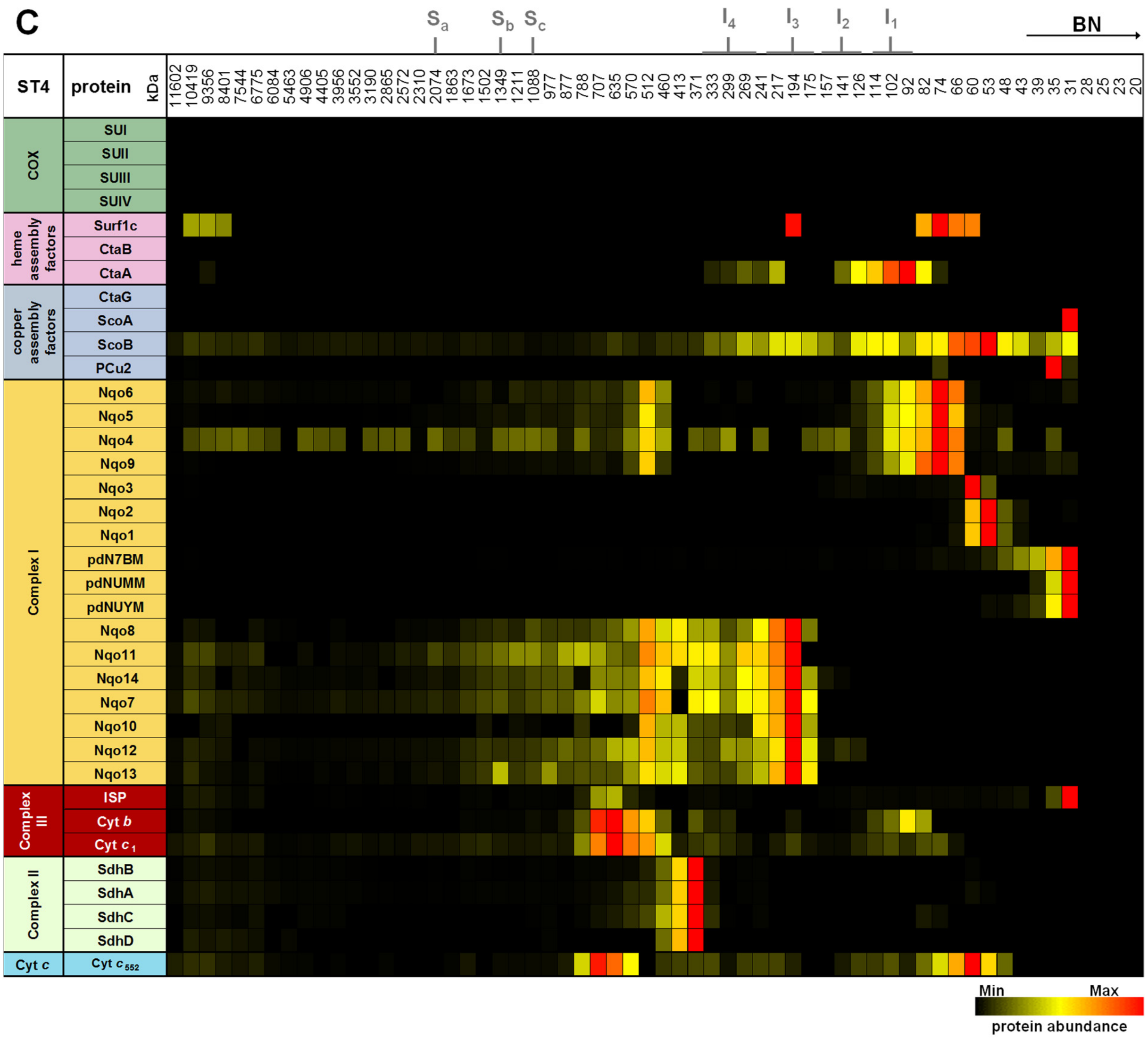

S3 Fig. Complexome profiles obtained from *P. denitrificans* WT and deletion strains. Membranes (140 µg) obtained from *P. denitrificans* WT (A) and deletion strains MR31 (B) and ST4 (C) were solublized with digitionin with a detergent/protein ratio of 2 g/g and separated by BN-PAGE. The gel lanes were cut into 60 identical slices, trypsin digested and analyzed by liquid chromatography tandem-mass spectrometry. The complexome profiles of respiratory chain complexes and oxidase assembly factors are shown as heat map illustration. For each identified protein, intensity-based absolute quantification (IBAQ) values were normalized to maximum appearance over all 60 slices. Oxidative phosphorylation complexes from bovine heart mitochondria were used for mass calibration. The position of supercomplexes S_{a-c} and assembly intermediates I_{1-4}, as found in the WT strain, is indicated (black bars in WT, grey bars in strain MR31 and ST4).
