# Supplementary material for: Cytochrome c Oxidase Biogenesis and Metallochaperone Interactions: Steps in the Assembly Pathway of a Bacterial Complex
Source: PLoS One. 2017 Jan 20;12(1):e0170037. doi: 10.1371/journal.pone.0170037 (PMC5249081; doi:10.1371/journal.pone.0170037)
Supplement: S4 Fig — Digitonin-solubilized membranes (140 μg) from P. denitrificans were separated by BN-PAGE and subsequently analyzed by complexome profiling. The average relative abundance value of each complex (taking all its subunits into account) is plotted against the apparent molecular mass in the BN gel. All profiles are coloured as shown in the legend. (A) The migration pattern of respiratory chain complexes I, III and IV, as well as of cyt c552 is shown in a WT background. Co-migration in the supercomplexes Sa-c is indicated, where complex I is only present in supercomplex Sa. Membranes from strain MR31 (B) or strain ST4 (C) do not exhibit supercomplexes. (PDF) [file pone.0170037.s004.pdf]

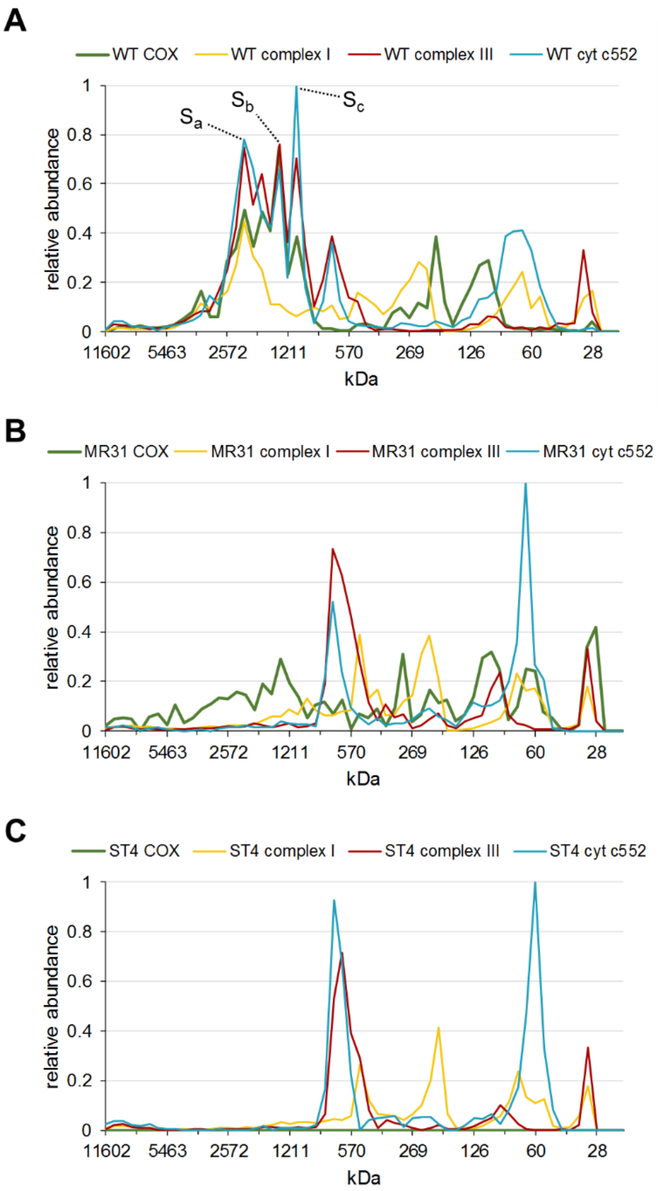

S4 Fig. Migration profiles of respiratory chain complexes. Digitonin-solubilized membranes (140 µg) from *P. denitrificans* were separated by BN-PAGE and subsequently analyzed by complexome profiling. The average relative abundance value of each complex (taking all its subunits into account) is plotted against the apparent molecular mass in the BN gel. All profiles are coloured as shown in the legend. (A) The migration pattern of respiratory chain complexes I, III and IV, as well as of cyt c_{552} is shown in a WT background. Co-migration in the supercomplexes $S_{a\text{-}c}$ is indicated, where complex I is only present in supercomplex S_a. Membranes from strain MR31 (B) or strain ST4 (C) do not exhibit supercomplexes.
